# SARS-CoV-2 uses major endothelial integrin αvβ3 to cause vascular dysregulation *in-vitro* during COVID-19

**DOI:** 10.1371/journal.pone.0253347

**Published:** 2021-06-23

**Authors:** Danielle Nader, Nicola Fletcher, Gerard F. Curley, Steven W. Kerrigan

**Affiliations:** 1 Cardiovascular Infection Research Group, School of Pharmacy and Biomolecular Sciences, RCSI University of Medicine and Health Sciences, Royal College of Surgeons in Ireland, Dublin 2, Ireland; 2 School of Veterinary Medicine, Veterinary Science Centre, University College Dublin, Belfield, Dublin 4, Ireland; 3 Department of Anaesthesia and Critical Care Medicine, RCSI University of Medicine and Health Sciences, Royal College of Surgeons in Ireland, Beaumont Hospital, Dublin, Ireland; Hungarian Academy of Sciences, HUNGARY

## Abstract

The unprecedented global COVID-19 pandemic has prompted a desperate international effort to accelerate the development of anti-viral candidates. For unknown reasons, COVID-19 infections are associated with adverse cardiovascular complications, implicating that vascular endothelial cells are essential in viral propagation. The etiological pathogen, SARS-CoV-2, has a higher reproductive number and infection rate than its predecessors, indicating it possesses novel characteristics that infers enhanced transmissibility. A unique K403R spike protein substitution encodes an Arg-Gly-Asp (RGD) motif, introducing a potential role for RGD-binding host integrins. Integrin αVβ3 is widely expressed across the host, particularly in the endothelium, which acts as the final barrier before microbial entry into the bloodstream. This mutagenesis creates an additional binding site, which may be sufficient to increase SARS-CoV-2 pathogenicity. Here, we investigate how SARS-CoV-2 passes from the epithelium to endothelium, the effects of αVβ3 antagonist, Cilengitide, on viral adhesion, vasculature permeability and leakage, and also report on a simulated interaction between the viral and host protein *in-silico*.

## Introduction

The unprecedented and ongoing Coronavirus disease 2019 (COVID-19) pandemic has caused a severe shock to the entire global population. As the global death toll continues to rise, vaccine candidates and targeted, effective anti-viral and immunotherapies are desperately sought after. Compared with diseases from other coronaviruses, COVID-19 has more adverse effects on the cardiovascular system. High rates of deep vein thrombosis and pulmonary embolism have been associated with severe SARS-CoV-2 infection [1], while COVID-19 presentations with stroke, myocardial infarction and disseminated intravascular coagulopathy have been reported [2, 3]. In addition, accumulating evidence suggests that microvascular occlusion within the lungs plays an important role in COVID-19 pathogenesis. Post-mortem studies have demonstrated widespread microthrombi throughout the pulmonary vasculature in patients with fatal COVID-19 [4], and right ventricular dysfunction in critically ill COVID-19 patients is common [5]. Data from autopsy studies in COVID-19 have identified marked endothelial cell apoptosis, together with loss of tight junction integrity in the pulmonary microvasculature [4], while electron microscopy studies have shown SARS-CoV-2 viral particles within pulmonary endothelial cell, suggesting that direct pulmonary endothelial cell infection may be important in triggering COVID-19 associated vasculopathy [6]. Vascular endothelial cells are essential in viral propagation, yet the mechanistic understanding behind this pathway remains elusive. The etiological pathogen of COVID-19, severe acute respiratory syndrome coronavirus 2 (SARS-CoV-2), is far more transmissible than its coronavirus predecessors [7]. It is clear that SARS-CoV-2 possesses a new characteristic not shared with previous coronaviruses, which has resulted in a widespread pandemic. Studies have established that the SARS-CoV-2 spike protein is significantly involved in host recognition and viral attachment, and interacts with the host receptor angiotensin-converting enzyme 2 (ACE2) through its receptor binding domain [8]. However, a novel K403R mutation in the spike protein of SARS-CoV-2 forms a unique Arg-Gly-Asp (RGD) motif outside the ACE2 recognition site. This introduces a potential role for RGD-binding integrins in viral entry through the spike protein [9, 10]. The endothelial barrier is highly populated with these integrins, where the major endothelial cell integrin, αVβ3, is capable of recognising a plethora of RGD-containing ligands. It contains a binding pocket that interacts with various proteins across the extracellular matrix, including fibrinogen, fibronectin, and vitronectin, which function to regulate adhesion, cellular migration, proliferation and angiogenesis. By developing this evolutionary mutation which provides the spike protein with an additional binding site, it may be sufficient to increase SARS-CoV-2 pathogenicity by enhancing viral attachment to host and causing higher transmissibility rates. Additionally, both ACE2 and αVβ3 integrin receptors are abundantly present across the host, providing several possible routes of entry for the virus whilst promoting dissemination across the host through a dual-receptor mechanism. Here we demonstrate that upon binding to the human vasculature, SARS-CoV-2 causes significant endothelial dysregulation resulting in loss of barrier integrity promoting shock and dissemination of secondary infection to major organs. This injurious effect inflicted by the virus could be significantly reduced by preventing the interaction with the major endothelial cell integrin αVβ3.

## Methods

### Cell and virus culture conditions

The human colonic cell line (Caco-2; ATCC^®^ HTB-37) were maintained in DMEM High Glucose media supplemented with 10% Fetal Bovine Serum, 1% Penicillin and Streptomycin. Primary derived Human Aortic Endothelial Cells (HAoEC; Promocell C-12271) were maintained in Endothelial Cell Media MV (PromoCell) supplemented with 10,000 U/mL Penicillin and 100 mg/mL Streptomycin. HAoEC were subject to shear hemodynamic force of 10 dynes/cm^2^ to mimic the physiological conditions of vascular stress. Human 2019-nCoV strain 2019-nCoV/Italy-INMI1 was obtained from the European Virus Archive Global (Ref. no: 008V-03893) and was used throughout the duration of this study at a Multiplicity of Infection (MOI) of 0.4 with viral titer at 1.08 x 10^5^ TCID50/mL.

### Para-nitrophenyl phosphate binding assay

SARS-CoV-2 interaction with Caco-2 and HAoEC was assessed using a binding assay utilising the fluorescent substrate, Para-Nitrophenyl Phosphate (pNPP). Inactivated virus aliquots were immobilised onto 96-well plates for 2 hours at 37°C, followed by blocking with 1% Bovine Serum Albumin (BSA). Cells were seeded for 2 hours at 37°C. Treated cells were administered with 0.05, 0.005, and 0.0005 μM of the αVβ3 antagonist, Cilengitide, (gift from H. Kessler, Technical University of Munchen, Germany) for 1 hour. Cells were added onto immobilised SARS-CoV-2 and allowed to adhere for 2 hours. A lysis buffer (0.1M NaOAc, 15mM pNPP, 0.1% TritonX-100, pH = 5.5) was added, containing the fluorescent marker para-nitrophenyl phosphate, which acts as a substrate for intracellular alkaline phosphatase. The reaction was stopped using 1M NaOH and pNPP activity was read at 405 nm in an automated plate reader (Victor, Perkins-Elmer). Following cell lysis, the resulting fluorescent signal was measured through absorbance at 405 nm. Binding (%) was analysed relative to the untreated cells.

### Transwell permeability assay

Endothelial barrier injury of Caco-2 or sheared HAoEC was evaluated using a transwell permeability assay. Treated cells seeded onto top chambers received 0.0005μM Cilengitide for 1 hour, and following SARS-CoV-2 infection for 24 hours, Fluorescein isothiocyanate-dextran (250ug/mL, 40kDa, Sigma-Aldrich) was added to the endothelial cells. Permeability was assessed by quantifying the levels of FITC-Dextran that had passed into the lower chamber. The fluorescent intensity was measured with excitation and emission wavelengths of 490 and 520nm, respectively.

### Immunofluorescence microscopy

VE-Cadherin (Vascular endothelial cadherin) expression on sheared HAoEC was measured by immunofluorescence microscopy. Sheared HAoEC were immobilised on glass slides, followed by SARS-CoV-2 infection for 24 hours. Cells were subsequently stained using anti-VE-Cadherin mouse monoclonal IgG1 antibody, conjugated to AlexaFluor 488 (1:100, F-8 sc-9989, SantaCruz Biotechnology), and 4,6-diamidino-2-phenylindole in fluorescent mounting medium (Invitrogen). Treated cells were subject to 0.0005μM Cilengitide for 1 hour. Images were acquired using an AxioObserver Z1 microscope (Zeiss). Levels of VE-Cadherin were computed by measuring fluorescent intensity of cells subtracted from background in ImageJ software (U. S. National Institutes of Health).

### Fluorescent-based protein interaction assay

Interactions between the SARS-CoV-2 Spike protein and endothelial αVβ3 were quantified using a fluorescent-based protein assay. Plates were coated with 30 μg/mL of Recombinant Human Novel Coronavirus Spike protein (Cat. No: CSB-MP3324GMYSP, Date of purchase: 18/05/20, Cusabio) for 2 hours at 37°C. Plates were washed three times in PBS and blocked with 1% BSA for 2 hours at 37°C. Plates were washed as described, and 50 μg/mL of Recombinant Human Integrin alpha V beta 3 protein were added for 2 hours at 37°C. After washing, plates were incubated with AlexaFluor 488 conjugated anti-Integrin αVβ3 clone LM609 (MAB1976, Merck) diluted 1:100 in reagent diluent. After 1 hour, LM609 activity was measured at 450nm with an automated plate reader.

### Integrin-Spike protein structure modelling

The 3D structures of SARS-CoV-2 spike protein, SARS-CoV spike protein, and integrin αVβ3 in complex with RGD ligand were obtained from the RCSB Protein Data Bank (PDB ID: 6M0J, 5XLR, and 1L5G, respectively). FASTA sequences were retrieved from NCBI. The AMBER10:EHT force field in MOE was used to energy minimise the complexes to resolve the intramolecular interactions and remove modelling bias before and after docking. The SCWRL algorithm and AMBER2 force field were used to obtain binding affinity through the molecular dynamics simulation function in Yasara. All structures and figures were visualised and prepared using Molecular Operating Environment (MOE) and PyMol.

### Statistical analysis

Data are presented as mean ± standard error of the mean. Experiments were carried out in triplicate with a minimum of three independent experiments. Statistical differences between groups were assessed by ANOVA with Dunnett’s post hoc tests or t-tests, as indicated. P-value < 0.05 was considered to be significant. Asterisks indicate P-values; *P<0.05; **P<0.01; ***P<0.001; ****P<0.0001.

## Results

### Paracellular transport through human epithelial cells promotes entry of SARS-CoV-2 into the extracellular matrix

**Fig 1:**
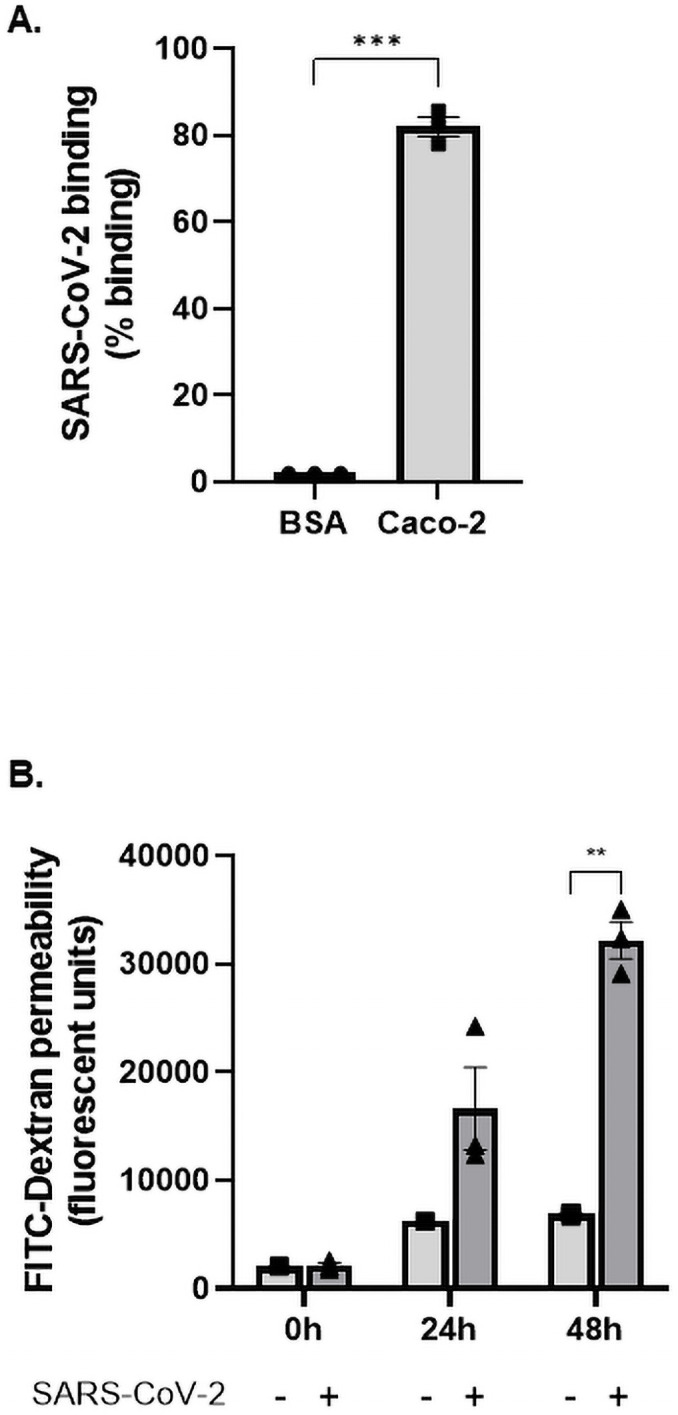
SARS-CoV-2 binds to human epithelial cells and causes permeability. (A) SARS-CoV-2 (1.08 x 10^5^ TCID50/mL) was added to either a control surface (BSA) or human epithelial cells (Caco 2). Cells were allowed to adhere to immobilised SARS-CoV-2 and lysed with pNPP, a fluorescent substrate against alkaline phosphatase expressed within cells. The fluorescent signal emitted by pNPP correlated to the number of cells adhered and was read at 405 nm. Epithelial cells significantly interacted with SARS-CoV-2 (P<0.001; paired t-test, N = 3). (B) Caco-2 was seeded onto transwell inserts and infected with SARS-CoV-2 at MOI = 0.4. Permeability was measured using Fluorescein isothiocyanate-dextran (FITC-Dextran, 40kDa) across 0 hours, 24 hours, and 48 hours. FITC-Dextran passes through the epithelial cells into the lower chamber and is proportionate to their permeability. The extent of permeability was measured by quantifying the fluorescent levels of FITC-Dextran at 490/520 nm. Cell permeability significantly increased throughout the time course (P<0.01; P<0.0001; ANOVA f-value = 46.84, N = 3).

Histopathological data has confirmed SARS-CoV-2 actively targets and infects human epithelial cells, resulting in severe cytopathic effects [11]. These primary manifestations commonly experienced in the lungs of COVID-19 patients include diffuse alveolar damage, tracheobronchitis, and interestingly, vascular injury [11, 12]. This suggests a method in which SARS-CoV-2 is capable of gaining access to the endothelium, located below the epithelial layer and interstitial space. Although virus-induced apoptosis and cytokine upregulation are consistently documented *in-vitro*, perhaps an additional route exists by which SARS-CoV-2 could traverse across the epithelium. High expression of Tumor Necrosis Factor (TNF) has been widely reported in patients with COVID-19, where increased levels of this pro-inflammatory cytokine promotes intestinal epithelial barrier permeability [13, 14]. This disruption of intercellular junctions encourages a paracellular mechanism for viral invasion similar to that found in other viral infections such as Dengue virus and Human Immunodeficiency Virus (HIV) [15, 16]. Consistent with this, we have demonstrated that SARS-CoV-2 binds strongly to epithelial cells (Fig 1A) and upon engagement results in loss of epithelial barrier formation, which increases significantly over 48 hours of infection (Fig 1B). This traversing facilitates dissemination of the virus into the interstitial space providing a mechanism through which it can gain entry to circulation.

### *In-silico* SARS-CoV-2 and endothelial αVβ3 complex reveals new potential binding pocket

**Fig 2:**
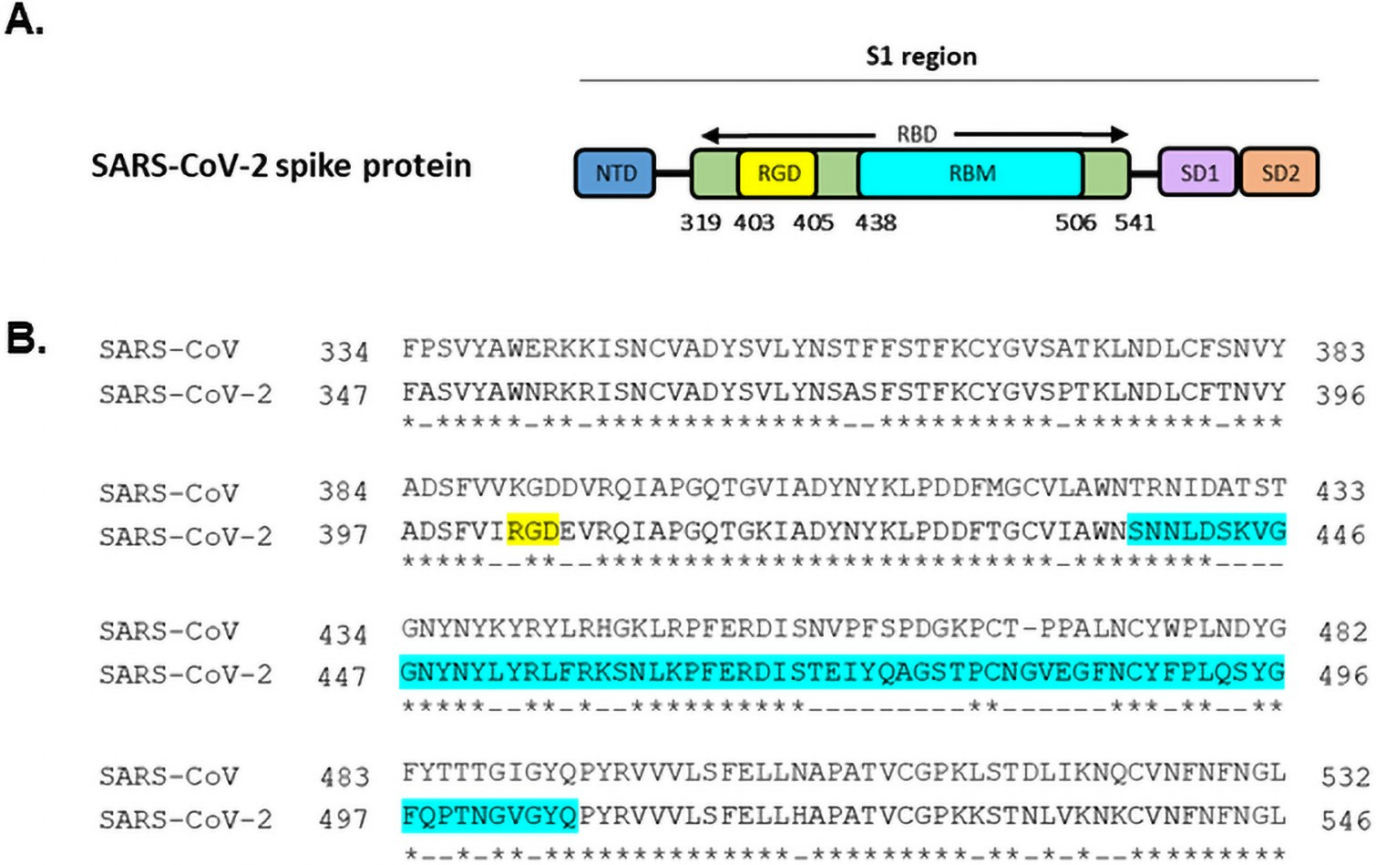
Sequence comparison between SARS-CoV and SARS-CoV-2 highlights the novel K403R mutagenesis. (A) Overall schematic drawing of the SARS-CoV-2 spike protein shows the N-terminal domain (NTD), RGD motif (Arg-Gly-Asp), receptor binding domain (RBD), receptor binding motif (RBM), sub-domain 1 (SD1), sub-domain 2 (SD2). The RGD motif resides within the receptor binding domain but adjacent to the ACE2-binding region (RBM). (B) Pairwise sequence alignment using EMBL-EMBOSS Needle contrasts the spike protein of SARS-CoV and SARS-CoV-2. Identical amino acids are signified by (*), dissimilar amino acids are signified by (-), RGD motif (yellow), RBM region (blue).

Cumulative data suggests that the enhanced transmissibility and infectivity of SARS-CoV-2 is attributed to mutations on the spike protein. Sequence analysis reveals a novel mutation absent in all coronavirus predecessors (Fig 2A and 2B). This presents evidence that this motif is not a universal feature of betacoronavirus ancestors. A point mutation at K403R introduces a widely recognised integrin recognition motif, Arginine-Glycine-Aspartic acid (RGD), into the spike protein. Ligands that contain the RGD motif bind to the major human endothelial integrin, αVβ3, with high affinity [17, 18]. Strikingly, this evolutionary motif resides nearby but not within the ACE2 binding site. Therefore alongside ACE2, the spike protein may also exploit a second human receptor. This would not only provide a more secure attachment onto host tissue, but also would ease SARS-CoV-2 dissemination between cells that simultaneously express both receptors. Significant evidence in the literature has demonstrated that other etiological agents of sepsis, *Staphylococcus aureus* and *Escherichia coli*, exploit this RGD-dependent mechanism to bind to host tissue via the major integrin αVβ3 expressed on the vascular endothelium [9, 19–21].

**Fig 3:**
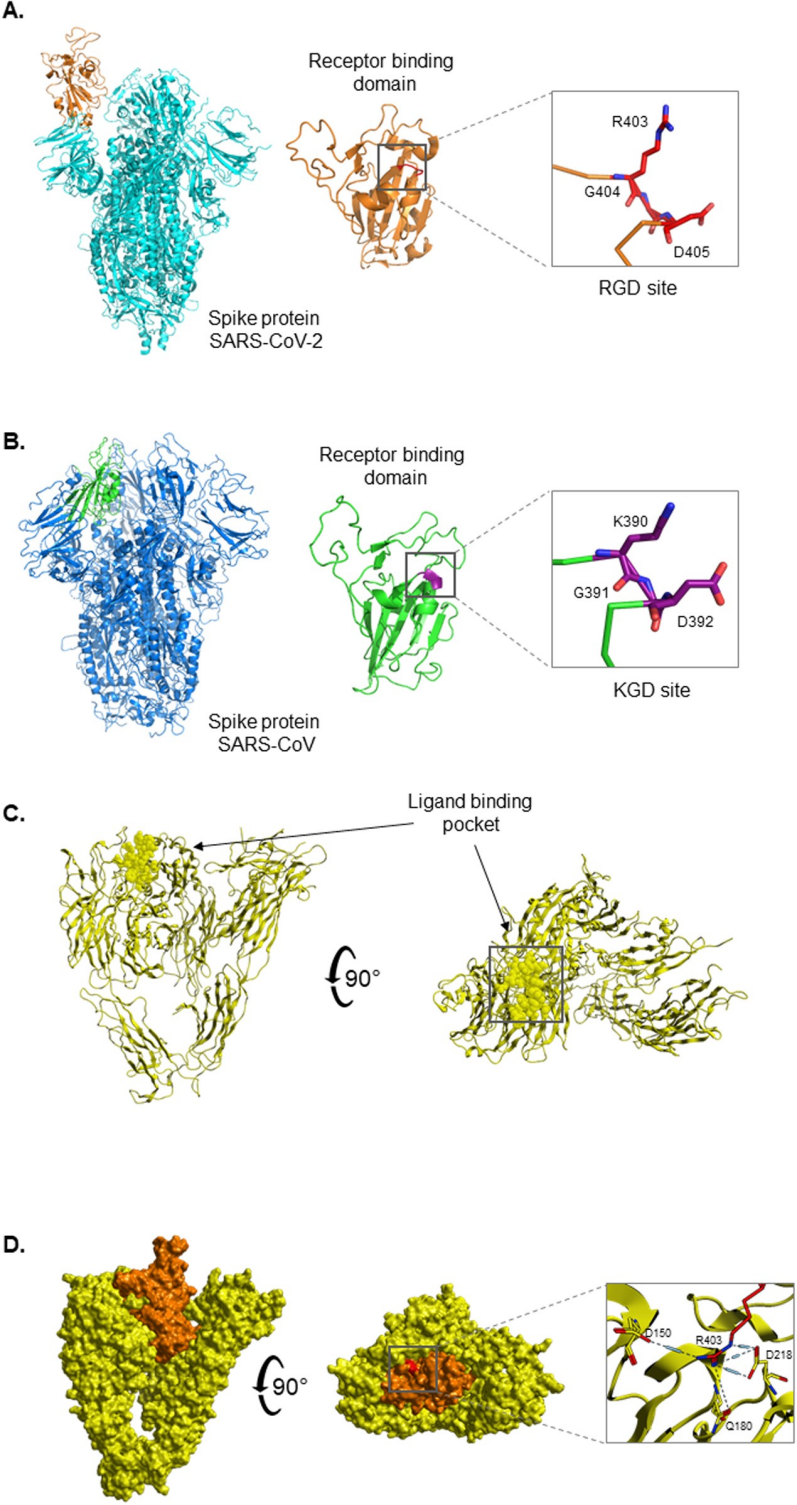
In-silico molecular interactions of SARS-CoV-2 spike protein with integrin αVβ3. (A) Ribbon representation of SARS-CoV-2 spike protein (orange) and receptor binding domain (cyan) (PDB ID 6M0J) with RGD motif rendered as stick (red). (B) Ribbon representation of SARS-CoV spike protein (blue) and receptor binding domain (green) (PDB ID 5XLR) with KGD residues rendered as stick (purple). (C) Ribbon representation of integrin αVβ3 (yellow) with ligand-binding pocket rendered in space-fill mode. (D) Docking the integrin αVβ3 (yellow) to receptor binding domain of SARS-CoV-2 spike protein (orange). A 90° orientation is also shown to visualise top view of the complex which highlights where the RGD motif sits (red). Integrin αVβ3 –SARS-CoV-2 interaction surface displaying contact points with RGD motif. Protein structures were constructed using PyMol.

To understand the structural interaction between SARS-CoV-2 and αVβ3, we constructed an *in-silico* molecular simulation of the theoretical complex. We first compared the receptor binding domains of spike proteins between SARS-CoV-2 and SARS-CoV. Analysis of these structures confirmed the presence of a point mutation at residue 403, substituting lysine for arginine. G404 and D405 were conserved between SARS-CoV-2 and SARS-CoV. Interestingly, both KGD and RGD sites are part of long, flexible loops. However, the RGD motif of SARS-CoV-2 is more solvent-exposed and therefore more likely to interact with neighbouring amino acids, in contrast to the KGD residues in SARS-CoV which do not appear to be as exposed to the solvent (Fig 3A and 3B). Following this, we docked the receptor binding domain of SARS-CoV-2 into the ligand-binding pocket within αVβ3. Interestingly, the RGD motif within the spike protein docks perfectly to the ligand binding pocket of αVβ3, and does not overlap with the ACE2 binding site. The RGD site is surface accessible within the receptor binding domain of the spike protein and is within the correct conformation to interact with the host cell. This signifies that the structural features of SARS-CoV-2 spike protein are highly compatible with its unique new second target–the αVβ3 integrin. The interaction of SARS-CoV receptor binding domain with αVβ3 was also investigated. MOE interaction energy maps revealed the mutagenesis that occurred from lysine to arginine resulted in fewer contact points with the integrin receptor. The glycine and aspartic acid residues in both SARS-CoV and SARS-CoV-2 retained conserved interactions with the integrin. However, the substitution from lysine to arginine resulted in additional contact points to D150 and Q180 with the integrin β chain. The predicted binding energy between R403 and D218 was significantly stronger in SARS-CoV-2 than SARS-CoV (-31.19 kcal/mol and -1.9 kcal/mol, respectively). In addition, the binding affinity for both in-silico complexes was calculated using Yasara. SARS-CoV-2 displayed a 400,000 kcal/mol increase in binding affinity for the integrin receptor than SARS-CoV (-1.8 × 106 kcal/mol and -1.4 × 106 kcal/mol). Therefore, the Lys/Arg difference could be attributed to the higher affinity of SARS-CoV-2 for αVβ3.

### SARS-CoV-2 interacts with human vascular endothelial cells through an evolutionary RGD mutation

**Fig 4:**
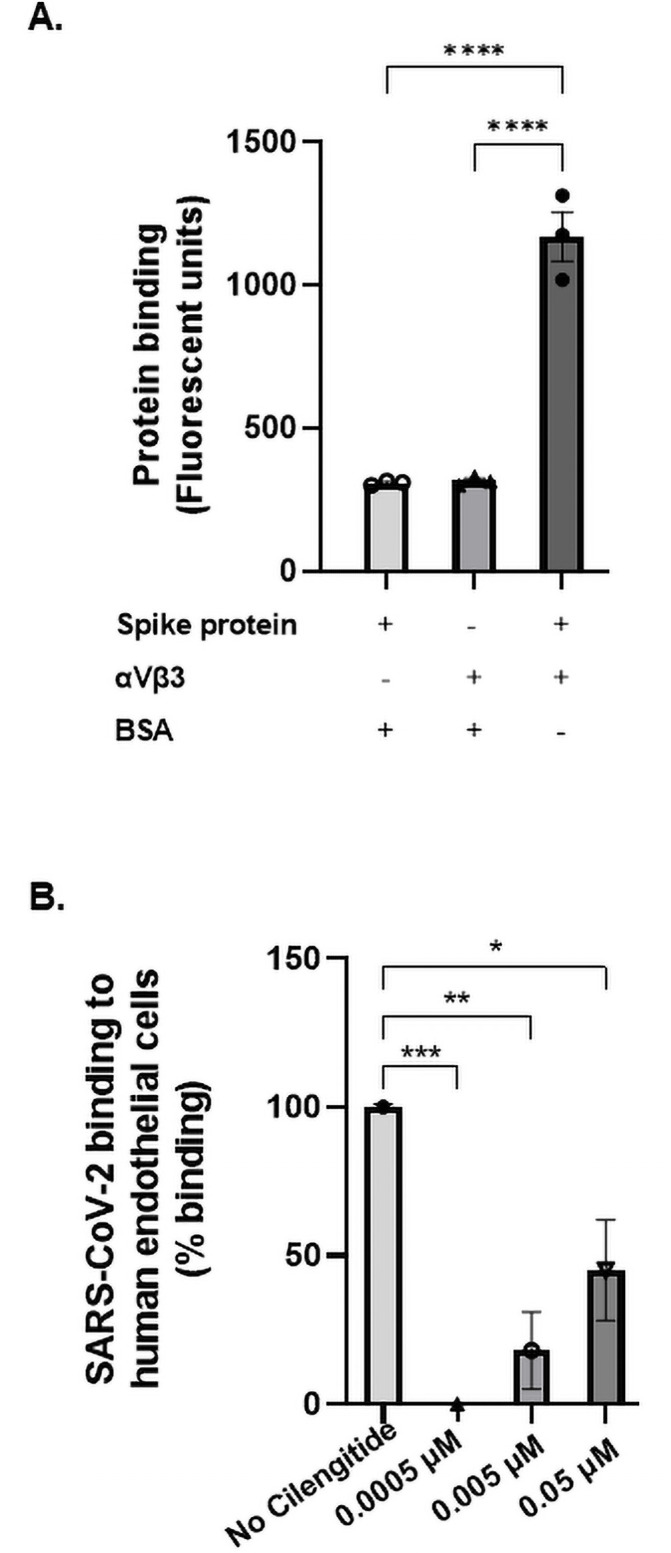
The RGD motif of SARS-CoV-2 spike protein mediates interaction to human endothelial cells. (A) The interaction between purified viral spike protein and endothelial integrin protein was assessed through an immunofluorescence binding assay. Recombinant SARS-CoV-2 spike protein was immobilised and its binding to recombinant αVβ3 protein was measured using an anti-αVβ3 fluorescent antibody (LM609-AF488, 1:100). Binding was analysed by measuring absorbance at 450 nm. The spike protein bound to αVβ3 showed significant interaction (P<0.0001; ANOVA f-value = 99.94, N = 3). (B) An in-vitro infection model investigated the adhesion potential of SARS-CoV-2 (1.08 x 10^5^ TCID50/mL) to endothelial cells. Sheared human aortic endothelial cells were activated with the cytokine TNFα to induce a pro-inflammatory state similar to that experienced in COVID-19 sepsis. Cilengitide was administered in 10-fold increments (0.05 μM– 0.0005 μM). A pNPP binding assay indicated % binding according to fluorescent signal measured at 405 nm. Statistical significance was found between the no drug control and each Cilengitide treated group. Since 0.0005 μM eliminated the host-viral interaction, it was chosen as optimal concentration for subsequent experiments (P = 0.001, ANOVA f-value = 16.58, N = 3).

Binding both ACE2 and αVβ3, which are respectively major epithelial and endothelial receptors, could explain how SARS-CoV-2 has expanded its tissue range of infection. We investigated this matter by examining the interactions between purified and tagged spike protein and human endothelial αVβ3, and found SARS-CoV-2 spike protein binds strongly to αVβ3 (Fig 4A). Furthermore, the *ex-vivo* infection model indicated SARS-CoV-2 attaches to human vascular endothelial cells in the presence of activation marker, TNF-α. A remarkable finding revealed the spike protein-αVβ3 interaction can be blocked by the specific αVβ3-antagonist Cilengitide, a tripeptide comprised of Arg-Gly-Asp residues, in an inverse dose-dependent manner (Fig 4B). The αVβ3 antagonistic molecule binds with high affinity to αVβ3 and is able to eliminate the interaction between the virus and the endothelium when used at 0.0005 μM.

### Vascular permeability and disruption of intercellular junctions induced by SARS-CoV-2 can be prevented by blocking host αVβ3 receptor

**Fig 5:**
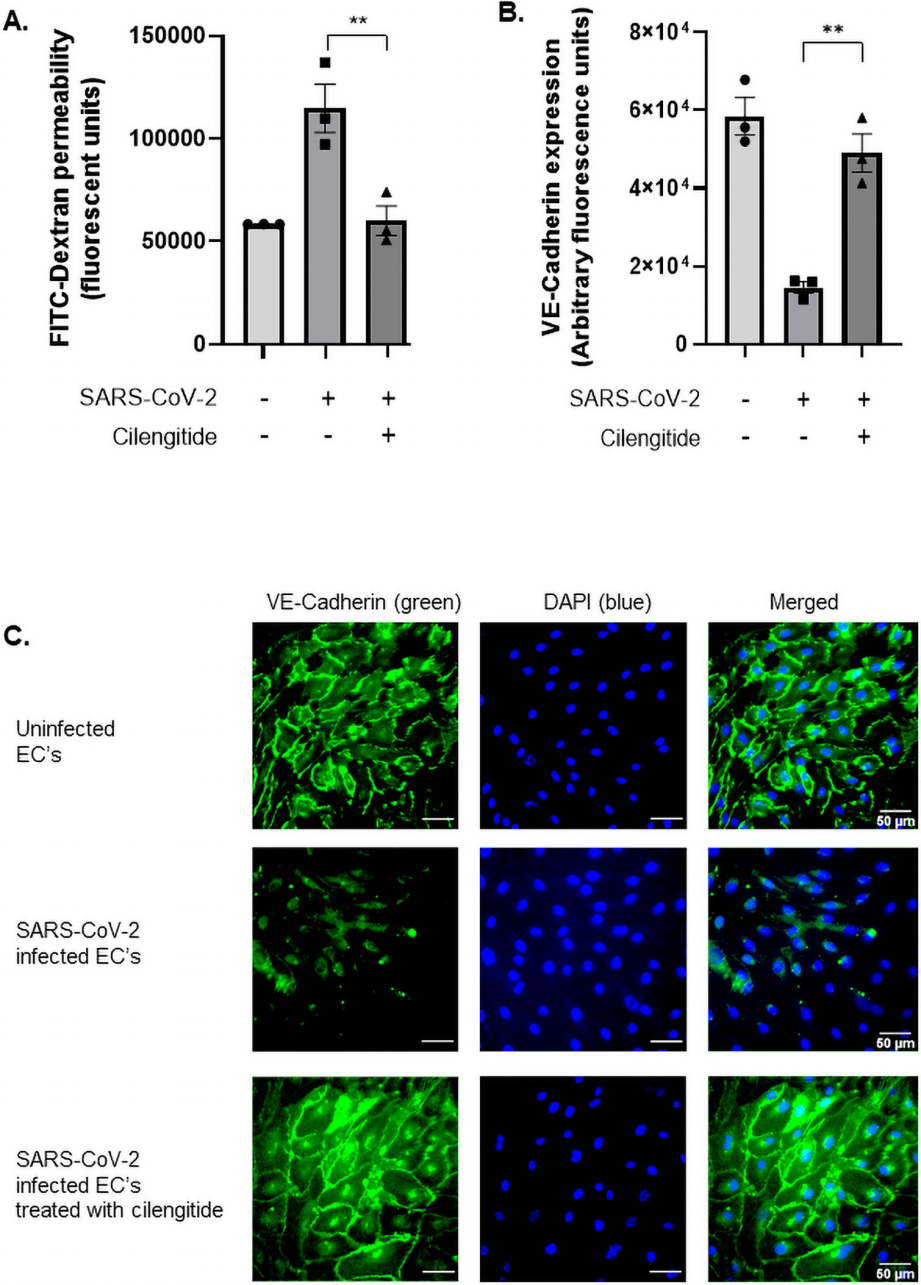
Vascular dysregulation occurs during SARS-CoV-2 infection and can be prevented using an αVβ3 antagonist, Cilengitide. (A) Both treated and untreated endothelial cells were seeded, sheared, and activated in transwell inserts to simulate the physiological conditions of blood flow and stress experienced by the vasculature in-vivo. Following Cilengitide treatment (0.0005 μM) for one hour, cells were inoculated with SARS-CoV-2 (1.08 x 10^5^ TCID50/mL) for 24 hours. Fluorescein isothiocyanate-dextran (FITC-Dextran, 40kDa) was added, which passed through the endothelial cell monolayer into the lower chamber, proportionate to the barrier’s permeability. The extent of permeability was measured by quantifying the fluorescent levels of FITC-Dextran at 490/520 nm. The significant rise in permeability levels indicated severe loss of barrier integrity following SARS-CoV-2 infection, but was restored after treating cells with Cilengitide (P = 0.005; ANOVA f-value = 16.27, N = 3). (B) VE-cadherin levels in uninfected, infected, and treated cells were assessed as a marker of barrier integrity and quantified based on mean fluorescence. The vasculature experienced severe vessel leakage following viral infection, due to significantly reduced expression of VE-Cadherin. When treated with Cilengitide for one hour, barrier permeability was restored back to uninfected levels (P = 0.02; ANOVA f-value = 32.63, N = 3). (C) Vascular-endothelial cadherin (VE-Cadherin) expression on sheared, activated endothelial cells was assessed using immunofluorescence microscopy. Treated cells received 0.0005 μM Cilengitide. Infected cells were inoculated with SARS-CoV-2 for 24 hours. Cell junctions were stained with anti-VE Cadherin antibody (green) and 4,6-diamidino-2-phenylindole (blue) for nuclei staining. Scale bar at 50 μm. Uninfected cells were first visualised using fluorescent microscopy at magnification 43X (top panel). Infected endothelial cells experienced loss of monolayer connections, by reduced expressed of VE-Cadherin (middle panel). Blocking the viral RGD to host αVβ3 pathway with Cilengitide re-established the barrier integrity after 24 hours (bottom panel).

A primary manifestation of COVID-19 is the loss of endothelial barrier integrity, which promotes vascular leakage, tissue oedema, hypoxia, and also encourages the dissemination of the virus to major organs, contributing to multiple organ failure and ultimately, death. Vascular-endothelial cadherin (VE-cadherin) is a crucial tight junction protein that plays a key role in maintaining the intact endothelial layer. Addition of SARS-CoV-2 to human endothelial cells resulted in a significant increase in endothelial cell permeability suggesting a breakdown in barrier junctions (Fig 5A). This is a significant finding, as change in the endothelial morphology is likely accompanied by disruption of intercellular junctions and loss of contact as a whole monolayer. To investigate this we measured the loss of VE-cadherin through quantitative immunofluorescence. Uninfected human endothelial cells maintained a tight barrier formation. However, upon infection with SARS-CoV-2, VE-cadherin surface expression on human endothelial cells was significantly reduced, suggesting cell–cell detachment and loss of barrier integrity occurred following infection (Fig 5B and 5C). This likely occurred due to the activation of the β3 subunit through an RGD-containing ligand, here represented by the SARS-CoV-2 spike protein. Previous studies demonstrated that engagement of β3 results in VE-cadherin internalisation and increased vascular leakage [22, 23]. A striking observation was that Cilengitide was capable of inhibiting viral attachment to endothelial αVβ3, which in turn prevented VE-cadherin reduction during infection. It also limited the occurrence of barrier permeability, returning VE-cadherin levels back to uninfected levels (Fig 5B and 5C).

## Discussion

The endothelium is at the forefront of microbial attack and plays a critical role in securing the integrity of the monolayer whilst maintaining an active response to stress. In response to injury induced by a pathogen, the delicate endothelium balance is disrupted, tipping towards vasculature dysregulation. This triggered response may go uninterrupted and become excessive in the presence of a severe infection, hence resulting in sepsis. The frequent occurrence of sepsis-like symptoms in COVID-19 patients such as elevated lactate dehydrogenase, D-dimers, and cytokine storms indicates sepsis is a shared outcome [24]. In addition, 100% of COVID-19 non-survivors develop sepsis, where they experience systemic inflammation, microthrombosis, acute cardiovascular complications, and severe organ dysfunction [24, 25]. The pronounced inflammatory damage and injury caused by SARS-CoV-2 prompted us to further investigate the nature of its interaction with the endothelium and its role in disease severity.

Docking simulations carried out between the integrin and both coronaviruses revealed novel differences in the binding interface. A striking difference resides in the mutation from lysine to arginine at residue 403 within SARS-CoV-2. This substitution introduces a significantly stronger hydrogen bond with Asp218 in the integrin α chain. This demonstrates that the evolutionary RGD motif strengthens the SARS-CoV-2 spike protein and integrin receptor interface. We then investigated the contrasting biochemical properties of SARS-CoV K390 and SARS-CoV-2 R403. Both lysine and arginine frequently appear on protein surfaces because of their critical role in maintaining ligand to receptor contacts through hydrogen and electrostatic bonds [26]. The flexible glycine and bulky aspartic acid residues within the region of interest are shared in SARS-CoV and SARS-CoV-2 spike proteins (Fig 2). However, the guanidinium group in arginine creates more interactions with αVβ3, due to 3 asymmetric amines, in contrast to lysine, which retains a lone amine group. This results in more electrostatic points of contact between SARS-CoV-2 and αVβ3 moving the spike protein closer to its receptor [27, 28]. Furthermore, the high pKa of arginine offers more stable ionic bonds due to delocalization of the positive charge across the π bonds of guanidinium which results in more resonance forms [27, 28]. In comparison to the lysine residue of SARS-CoV, the conservational replacement with arginine at site 403 is likely to be more favourable for αVβ3 interactions.

Although ACE2 was identified as being the sole receptor involved with host attachment, entry and invasion, SARS-CoV-2 remains capable of targeting ACE2-negative colonic enterocytes and liver cells. This phenomenon is particularly significant as acute hepatic impairment is prominent in COVID-19 patients [29, 30]. RNA expression of ACE2 indicates high levels within the alveolar epithelium and gastrointestinal tract [31, 32]. In contrast, the integrin αVβ3 retains cytoplasmic and surface membrane expression in nearly all tissues and cells of mesenchymal origin, including gastrointestinal, respiratory, and urinary systems [32–34]. Additionally, αVβ3 has higher expression patterns across the alveolar and vascular endothelium than ACE2 [34, 35]. This finding illustrates the substantial benefits that SARS-CoV-2 has attained by potentially gaining a second host receptor. Most critically we identified that blocking αVβ3 significantly prevented SARS-CoV-2 from binding to the vascular endothelium. Consistent with previous observations cilengitide has a stabilizing effect on the vascular endothelium and is more effective at lower concentrations than higher concentrations (inverse dose response) which is likely due to it acting as a ligand at higher concentrations and therefore less effective [19].

Conclusively, SARS-CoV-2 binding to the epithelium creates a paracellular route of invasion by causing significant changes in permeability. Following this breakdown, the virus passes through the interstitial space and attaches to abluminally expressed αVβ3 on the vascular endothelium. This interaction is likely mediated through the evolutionary K403R motif within the spike protein that promotes binding to αVβ3. This mutagenesis could elucidate the enigma of the endothelial role during COVID-19 pathogenesis, where vascular dysregulation is a prominent occurrence in severely affected patients. The RGD motif has the potential to function in viral attachment and downstream pathogenic injury for SARS-CoV-2. We have also provided structural evidence at the molecular level to suggest that this motif resides in an appropriate site within the spike protein that functions in targeting dual host receptors. Additionally, we have observed that in all major SARS-CoV-2 variants, including the Clustal 5 and VUI 202012/01 variants, the RGD site is conserved, suggesting these coronaviruses maintain the ability to anchor to αVβ3 [36, 37]. This contrasts all coronavirus predecessors that lack the RGD motif. Finally, we have provided evidence that the addition of an αVβ3 antagonist, Cilengitide, significantly prevents both attachment to the host and disruption of barrier integrity. Despite these findings an important next step will be to identify the exact role of the ACE2 and αVβ3 receptors in the interaction using a transgenic animal model.

Introducing a potential therapeutic option is particularly relevant during COVID-19 treatment. Inhibiting the initial attachment of microbes is a highly effective method to prevent the development of sepsis which is triggered by pathogenic dissemination at the level of the endothelium. Blocking this interaction may significantly reduce the vascular dysregulation and cardiac dysfunction experienced by COVID-19 patients.

## Supporting information

S1 File (XLSX)
